# Association Between Vertical Bone Defects and Interdental Papilla Loss in Periodontitis: A Cross-Sectional Analysis

**DOI:** 10.3390/dj13070294

**Published:** 2025-06-29

**Authors:** Hristina Maynalovska, Kamen Kotsilkov

**Affiliations:** Department of Periodontology, Faculty of Dental Medicine, Medical University of Sofia, 1431 Sofia, Bulgaria; k.kotsilkov@fdm.mu-sofia.bg

**Keywords:** interdental papilla, vertical bone defects, loss of papilla height, periodontitis, “black triangles”

## Abstract

**Background:** The interdental papilla plays a critical role in maintaining both the esthetic and functional integrity of the periodontium. Although the relationship between the papilla presence and the contact point–bone crest distance is well established, the impact of vertical bone defect morphology—common in periodontitis—remains largely unexplored. **Aim:** To assess the relationship between the loss of interdental papilla height and three site-specific factors—vertical bone defect morphology, probing depth, and papilla base width—in patients with periodontitis. **Materials and Methods:** Ten periodontitis patients contributing 28 interdental papillae adjacent to vertical bone defects were included. The recorded parameters included probing depth, papilla base width, and loss of papilla height. Intraoperative measurements of defect depth, mesiodistal width, and buccolingual width were also obtained. Patient-level variables, such as age, sex, oral hygiene, and gingival phenotype, were not controlled or included in the analysis, due to the small number of participants and the study’s focus on defect-level characteristics. Spearman’s rank correlation was used due to non-normal data distribution. **Results:** A moderate positive association was observed between the probing depth and loss of papilla height (ρ = 0.353), approaching but not reaching statistical significance (*p* = 0.066). Weak, non-significant associations were found with the remaining parameters (*p* > 0.05). **Conclusions:** Although no statistically significant associations were found, observed trends may indicate site-specific influences on the loss of papilla height. These preliminary findings highlight the need for further research with larger, well-characterized cohorts to better understand the factors affecting papilla stability in periodontitis.

## 1. Introduction

The interdental papilla is a key component of the periodontium, essential for maintaining both the functional integrity and esthetic harmony of the dentition. Its loss can result in the formation of “black triangles,” phonetic disturbances, and increased food impaction, ultimately compromising patient satisfaction and periodontal health [1,2,3,4]. Open interdental spaces facilitate food impaction, which may lead to recurrent trauma, localized inflammation, and progressive periodontal breakdown. Vertical food impaction, typically resulting from the loss of proximal contacts or poorly contoured restorations, has been strongly associated with increased probing depths, bleeding on probing, and clinical attachment loss [5,6]. The persistent accumulation of food debris and bacterial biofilm in these spaces can initiate and sustain a cycle of inflammation that accelerates soft and hard tissue destruction [7,8].

The etiology of interdental papilla deficiency has been widely discussed in the literature, with numerous anatomical and clinical factors identified as contributing influences. One of the most critical determinants is the vertical distance from the alveolar bone crest to the base of the interproximal contact area. Tarnow et al. [9] demonstrated that when this distance exceeds 5 mm, the probability of complete papilla fill decreases significantly. Additional factors influencing papilla presence and stability include the dimensions and position of the interproximal contact area itself [10], the interproximal root distance [11,12], root angulation [13], crown morphology [14], the facio-lingual dimension of the embrasure space [15,16] and periodontal biotype [17].

Age-related reductions in the soft tissue volume and the progression of periodontal disease are also considered contributing factors [4,18].

Furthermore, changes in tooth positioning following orthodontic treatment can increase the risk of open gingival embrasures and incomplete papilla fill, as frequently reported in the literature [19,20].

Despite this broad understanding, most studies focus on healthy papillae, while relatively few have addressed the behavior of the papilla in the context of periodontal disease, where attachment and bone loss are present. Periodontal disease, characterized by chronic inflammation and progressive destruction of the supporting tissues, also plays a significant role in the interdental papilla loss. As the disease progresses, the breakdown of the alveolar bone and periodontal ligament results in apical migration of the gingival margin and the subsequent collapse of the interdental papillary architecture [21].

Among the various patterns of bone loss associated with periodontitis, vertical (angular) bone defects are of particular concern, as they create localized and often deeper osseous destruction adjacent to one or more teeth, critically impairing the soft tissue support [21,22].

Although many contributing factors have been identified, there is still limited understanding of how specific aspects of bone loss, such as the morphology and severity of vertical bone defects, affect the loss of interdental papilla height. This gap is particularly important in the context of periodontitis, where vertical bone defects are frequently observed [23], yet their specific impact on interdental soft tissue architecture remains insufficiently investigated. Most existing studies focus on intact periodontal sites [11,12,15,16,17].

The width of the papilla at its base was included as a site-specific soft tissue parameter, reflecting the size of the interproximal space and potentially influenced by the periodontal biotype. Broader papillae are typically associated with thicker tissue phenotypes, which may offer greater resistance to soft tissue loss, whereas narrower papillae in thinner biotypes may be more prone to recession, particularly when the contact point–bone crest distance is increased [4,18,24].

Accordingly, the aim of this study was to assess the relationship between the loss of interdental papilla height and three site-specific parameters: the morphology of vertical bone defects, probing depth at the defect site, and the width of the papilla at its base in periodontitis patients. We hypothesized that these localized clinical and anatomical features may be associated with increased papilla height loss in periodontally compromised sites.

## 2. Materials and Methods

### 2.1. Study Design

This cross-sectional observational study, based on a convenience sample of patients, aimed to evaluate the relationship between vertical bone defects and the interdental papilla status prior to surgical intervention. A total of 28 interdental papillae in 10 periodontitis patients were analyzed. The study was conducted at the Medical University of Sofia as part of a larger project investigating the clinical outcomes of minimally invasive surgical treatment using enamel matrix derivates and bone graft.

### 2.2. Eligibility Criteria

Patients eligible for inclusion were systemically healthy individuals aged 18 years or older, without known allergies, and diagnosed with Stage III or IV periodontitis according to the 2017 World Workshop classification [25]. Adequate oral hygiene was required, defined as a full mouth plaque score (FMPS) [26] and full mouth bleeding score (FMBS) [27] below 15% following initial periodontal therapy. In addition, participants had to present with at least one proximal angular bone defect characterized by a probing depth (PD) of ≥6 mm, bleeding on probing (BoP), and preserved interproximal contacts at the time of re-evaluation.

Patients were excluded if they had systemic conditions known to affect periodontal health (e.g., uncontrolled diabetes mellitus), required antibiotic prophylaxis for dental procedures, were current smokers, demonstrated insufficient plaque control, or were pregnant or breastfeeding [28,29].

At the tooth level, the inclusion criteria specified the presence of a proximal vertical bone defect with a radiographically confirmed intrabony component of ≥3 mm and a clinical probing depth of ≥6 mm, with no evidence of periapical pathology [30]. In addition, a minimum of 2 mm of keratinized tissue at the defect site was required, in accordance with the surgical protocol used [31]. Teeth were excluded if they exhibited inadequate endodontic treatment, periapical lesions, furcation involvement, Grade III mobility, were third molars, or were mispositioned or tilted.

### 2.3. Clinical and Surgical Measurements

A comprehensive periodontal examination was performed prior to surgical intervention. Probing depth (PD), clinical attachment level (CAL), bleeding on probing (BoP), and gingival recession (R) were recorded at six sites per tooth (mesiobuccal, buccal, distobuccal, mesiolingual, lingual, and distolingual) using a manual graduated periodontal probe (UNC-15, Hu-Friedy, Chicago, IL, USA). All data were systematically documented in standardized periodontal charts. The measurements were rounded to the nearest full millimeter.

All clinical measurements, including all analyzed dimensions of the interdental papilla, were performed by a single experienced examiner. To assess intra-examiner reliability, these measurements were recorded at two time points: one week before surgery and again on the day of the procedure. The comparison showed no significant differences between the two sets of values, confirming measurement consistency. Intraoperative measurements were performed only once during the surgical procedure, and were also conducted by the same examiner.

The assessment of the interdental soft tissues included the measurements of the papilla width at its base, the papilla height from the base to the tip, and the loss of papilla height. The base of the papilla was defined as the horizontal line connecting the emergence points of the adjacent teeth’s clinical crowns at the level of the gingival margin. The width of the papilla was measured along this line, corresponding to the base of the gingival embrasure. The loss of papilla height was defined as the vertical distance from the tip of the interdental papilla to the most apical point of the interproximal contact area between the adjacent teeth, measured using a periodontal probe positioned vertically along the long axis of the papilla [17,32].

At the time of surgical access, a minimally invasive surgical protocol was followed. A horizontal incision was placed at the base of the interdental papilla associated with the treated defect, complemented by a vertical incision extending to the marginal bone crest to allow direct access to the underlying bony structure. A full-thickness flap was carefully elevated, extending approximately 2 mm beyond the crestal bone margin to ensure adequate exposure of the defect. An additional incision was performed at the level of the intraosseous septum to gently separate the supracrestal soft tissues from those occupying the intrabony component of the defect [33].

Once access was achieved, measurements were obtained using a graduated periodontal probe. The depth of the intraosseous component was recorded as the distance from the base of the defect to the level of the interproximal bone septum of the adjacent tooth [34]. The buccolingual width was measured from the buccal to the lingual wall of the defect, and the mesiodistal width was recorded from the root surface to the opposing proximal bony wall.

### 2.4. Statistical Analysis

Statistical analyses were conducted using GraphPad Prism, version 9.5.1 (GraphPad Software, San Diego, CA, USA). The normality of data distribution was evaluated using the Shapiro- Wilk test. Spearman’s rank correlation coefficient was calculated to investigate the associations between the examined parameters. A correlation heatmap was generated using AI-based computational tools to visualize the strength and direction of the observed relationships.

The unit of analysis was the individual defect. Although multiple defects were included from some participants, each defect–papilla pair was considered a distinct and site-specific clinical entity. Given the small number of participants and the exploratory nature of the study, no statistical correction for within-subject clustering was applied. A *p*-value < 0.05 was considered statistically significant.

## 3. Results

The study population comprised 10 patients (3 males and 7 females), aged between 36 and 53 years (mean age: 42 ± 6 years), all diagnosed with Stage III or IV periodontitis. A total of 30 vertical bone defects were initially identified. Two defects were subsequently excluded due to their location on the distal surfaces of terminal teeth, where interdental papilla was absent. Consequently, 28 vertical bone defects were included in the final analysis.

In line with the aim of the study, the following parameters were evaluated: probing depth at the defect sites, width of the papilla at its base, loss of papilla height, and intraoperative measurements of bone defect depth, mesiodistal width, and buccolingual/palatal width.

The distribution of these variables was assessed using the Shapiro–Wilk test to determine whether parametric or non-parametric methods were appropriate for further analysis.

Due to the non-normal distribution observed in several variables, the relationships between the loss of papilla height and the other clinical and surgical parameters were evaluated using Spearman’s rank correlation coefficient.

The correlation analysis explored the association between the loss of papilla height and probing depth, papilla width at the base, bone defect depth, buccolingual width, and mesiodistal width.

**Figure 1:**
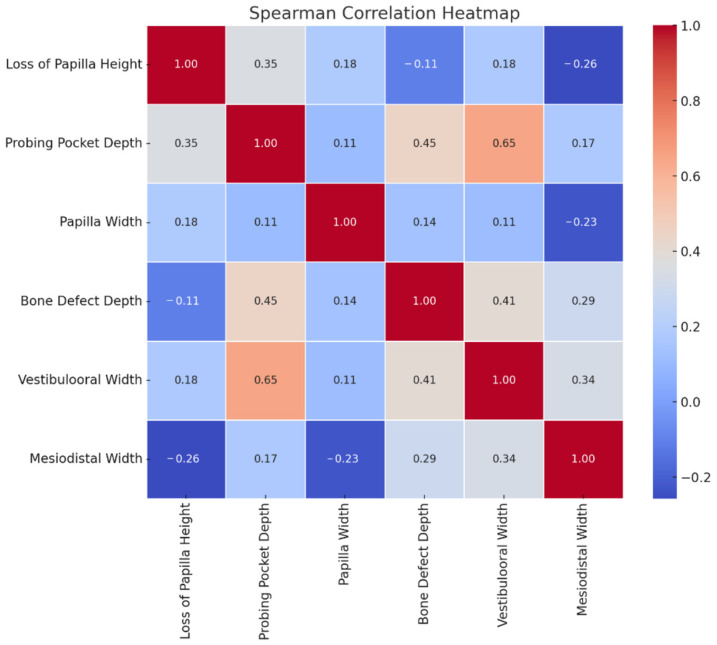
Correlation heatmap illustrating the Spearman’s rank correlation coefficients between the loss of papilla height and the measured clinical and surgical parameters.

The results of the Spearman’s correlation analysis are summarized in Table 1 and visualized in the correlation heatmap presented in Figure 1.

A weak positive association was observed between the loss of papilla height, the width of the papilla at its base (ρ = 0.184, *p* = 0.349), and the buccolingual width of the bone defect (ρ = 0.178, *p* = 0.365), indicating that greater papilla width and wider defects may be associated with increased papillary loss, although these relationships were not statistically significant.

The probing depth demonstrated the strongest correlation with papilla height loss (ρ = 0.353, *p* = 0.066), approaching but not reaching statistical significance.

Bone defect depth (ρ = –0.099, *p* = 0.616) and mesiodistal defect width (ρ = –0.258, *p* = 0.186) showed weak negative correlations with papilla height loss.

Overall, no statistically significant associations were identified among the parameters studied. Although weak to moderate correlations were observed among several parameters, none achieved statistical significance. These findings should therefore be interpreted with caution, and larger-scale studies are required to validate the observed trends.

## 4. Discussion

The current study investigated the relationship between the clinical characteristics of vertical bone defects and the associated loss of interdental papilla height in patients with advanced periodontitis. Despite evaluating key parameters, such as probing depth, defect morphology, and soft tissue dimensions, no statistically significant correlations were identified between the loss of papilla height and the measured variables.

The landmark study by Tarnow et al. [9] remains a cornerstone in our understanding of the anatomical factors governing the interdental papilla presence. By examining 288 sites across 30 patients, the authors demonstrated a clear inverse relationship between the vertical distance from the alveolar bone crest to the interproximal contact point and the presence of the papilla. Their data showed that when this distance was 5 mm or less, the papilla was nearly always intact. However, beyond this threshold—particularly at 6 mm or more—the frequency of papilla loss increased dramatically. These findings established that the height of the underlying bone plays a decisive role in papilla preservation.

While this relationship is well established in sites with an intact or only mildly compromised periodontium, considerably less is known about how the interdental papilla behaves in areas affected by advanced periodontal destruction, particularly in the presence of vertical (intrabony) bone defects. In the present study, we aimed to build upon the foundational insights of Tarnow et al. [9], Cho et al. [12], Chow et al. [24] and Joshi at al. [16] by examining not only the distance between the contact point and the alveolar bone crest, but also the specific morphological characteristics of angular defects and their relationship to papilla height. We hypothesized that these site-specific features could provide a more nuanced understanding of the clinical presentation of papilla loss in periodontitis-affected sites.

Unfortunately, the results of our analysis did not reveal any statistically significant associations between the examined parameters and loss of papilla height. A weak positive association was observed between the loss of papilla height and the buccolingual width of the vertical bone defect (ρ = 0.178, *p* = 0.365), suggesting a possible trend toward greater papilla loss in cases with wider osseous dimensions. Although these trends were not statistically significant, they may point to clinical patterns that warrant further investigation in larger cohorts.

In contrast, bone defect depth (ρ = –0.099, *p* = 0.616) and mesiodistal defect width (ρ = –0.258, *p* = 0.186) exhibited weak negative associations with papilla height loss. These results suggest that these dimensions may not be key determinants of papillary support, at least within the context and sample size of the present study.

The probing depth at the defect site demonstrated the strongest association with papilla height loss (ρ = 0.353, *p* = 0.066), approaching but not reaching statistical significance. This trend nonetheless supports previous findings that deeper periodontal pockets may contribute to the vertical collapse of the interdental tissues [35].

The width of the papilla at its base was included in our analysis as a clinically relevant parameter, reflecting the interproximal space and potentially influenced by periodontal biotype. Thicker biotypes are generally associated with broader, flatter papillae that are less prone to recession, while thinner biotypes tend to exhibit narrower, more tapered papillae with a higher risk of height loss, especially when the contact-to-bone distance increases [4,18,24,36]. However, previous findings by Barboza de Lemos et al. [37] suggest that at greater distances, thin biotypes may paradoxically be more favorable for papilla presence. In our study, a weak positive correlation was observed between the papilla base width and loss of papilla height (ρ = 0.184, *p* = 0.349), suggesting that wider papillae may not necessarily offer protection against soft tissue loss in the context of vertical bone defects. Although this correlation did not reach statistical significance, the observed trend highlights the need for further research to clarify the role of papilla base width in soft tissue stability around vertical defects.

This study has several limitations that must be acknowledged. First, the small sample size and the absence of a priori sample size calculation limit the statistical power of our findings. The analysis was based on a convenience sample of eligible cases, which may introduce selection bias. Furthermore, multiple defects were included from some participants, introducing the potential for intra-subject correlation. While we treated each defect–papilla pair as an anatomically and clinically distinct unit—consistent with the site-specific nature of periodontal disease—no statistical correction for clustering was applied, given the exploratory nature of the study.

Additionally, potential confounders such as age, sex, and oral hygiene were not included in the analysis. Due to the limited sample size and the focus on defect-level parameters, these patient-level variables were not considered as confounding factors. However, we recognize that such variables—as well as biological characteristics like the periodontal phenotype—may influence local soft tissue architecture and papilla stability, and their exclusion represents a methodological limitation.

Lastly, while the study centered on vertical defect morphology and its relationship to papillary loss, we acknowledge that additional variables—such as the distance from the contact point to the alveolar bone crest—are well-established in the literature and may provide valuable context. Although not included in this analysis, such parameters could be incorporated in future research to complement our findings and support broader clinical interpretations.

## 5. Conclusions

This cross-sectional study did not reveal statistically significant correlations between the papilla height reduction and the morphology of vertical bone defects in periodontitis patients. While some weak to moderate trends were observed, the multifactorial etiology of interdental papilla recession likely involves factors beyond the scope of these isolated clinical parameters. These results emphasize the complex nature of soft tissue dynamics in periodontal disease and necessitate further research employing alternative study parameters and larger patient populations. Moreover, additional radiographic analyses could be applied to assess contact point characteristics, the distances between the interdental alveolar bone crest, and both the cementoenamel junction (CEJ) and the contact point, as well as the width and configuration of the interproximal septum.

## Figures and Tables

**Table 1:** Results of the Spearman’s rank correlation analysis between the papilla height loss and other parameters.

Parameter	Spearman’s ρ	*p*-Value
Width at the base of the papilla (mm)	0.184	0.349
Probing depth (mm)	0.353	0.066
Bone defect depth (mm)	−0.099	0.616
Buccolingual width of bone defect (mm)	0.178	0.365
Mesiodistal width of bone defect (mm)	−0.258	0.186

## Data Availability

The data that support the findings of this study are available on request from the corresponding author.

## References

[1] Patel M., Guni A., Nibali L., Garcia-Sanchez R. (2024). Interdental papilla reconstruction: A systematic review. Clin. Oral Investig..

[2] Hochman M.N., Chu S.J., Tarnow D.P. (2012). Maxillary anterior papilla display during smiling: A clinical study of the interdental smile line. Int. J. Periodontics Restor. Dent..

[3] Cunliffe J., Pretty I. (2009). Patients’ ranking of interdental “black triangles” against other common aesthetic problems. Eur. J. Prosthodont. Restor. Dent..

[4] Zhang Y., Hong G., Zhang Y., Sasaki K., Wu H. (2020). Minimally invasive procedures for deficient interdental papillae: A review. J. Esthet. Restor. Dent..

[5] Hancock E.B., Mayo C.V., Schwab R.R., Wirthlin M.R. (1980). Influence of Interdental Contacts on Periodontal Status. J. Periodontol..

[6] Truong V.M., Kim S., Yi Y.J., Park Y.S. (2023). Food Impaction in Dentistry: Revisited. Oral Health Prev. Dent..

[7] Abdulkareem A.A., Al-Taweel F.B., Al-Sharqi A.J.B., Gul S.S., Sha A., Chapple I.L.C. (2023). Current concepts in the pathogenesis of periodontitis: From symbiosis to dysbiosis. J. Oral Microbiol..

[8] Könönen E., Gursoy M., Gursoy U. (2019). Periodontitis: A Multifaceted Disease of Tooth-Supporting Tissues. J. Clin. Med..

[9] Tarnow D.P., Magner A.W., Fletcher P. (1992). The Effect of the Distance From the Contact Point to the Crest of Bone on the Presence or Absence of the Interproximal Dental Papilla. J. Periodontol..

[10] Kolte A.P., Kolte R.A., Bawankar P. (2018). Proximal contact areas of maxillary anterior teeth and their influence on interdental papilla. Saudi Dent. J..

[11] Kolte A.P., Kolte R.A., Mishra P.R. (2014). Dimensional Influence of Interproximal Areas on Existence of Interdental Papillae. J. Periodontol..

[12] Cho H.S., Jang H.S., Kim D.K., Park J.C., Kim H.J., Choi S.H., Kim C.K., Kim B.O. (2006). The Effects of Interproximal Distance Between Roots on the Existence of Interdental Papillae According to the Distance From the Contact Point to the Alveolar Crest. J. Periodontol..

[13] Sharma A.A., Park J.H. (2010). Esthetic Considerations in Interdental Papilla: Remediation and Regeneration. J. Esthet. Restor. Dent..

[14] Nichani A., Jameel Ahmed A., Ranganath V. (2016). The Shape of the Maxillary Central Incisors and Its Correlation with Maxillary Anterior Papillary Display: A Clinical Study. Int. J. Periodontics Restor. Dent..

[15] Kim S.A., Choi S.S., Byun S.J., Chang M. (2011). Analysis of the embrasure dimensions between maxillary central incisors in relation to the topography of the interdental papilla. J. Periodontal Implant Sci..

[16] Joshi K., Baiju C.S., Khashu H., Bansal S., Maheswari I.B. (2017). Clinical assessment of interdental papilla competency parameters in the esthetic zone. J. Esthet. Restor. Dent..

[17] Khaireddine H., Mohamed T., Arij R., Faten K., Faten B.A. (2023). Factors impacting the height of the interproximal papilla: A cross-sectional study. Clin. Exp. Dent. Res..

[18] Montevecchi M., Checchi V., Piana L., Checchi L. (2011). Variables Affecting the Gingival Embrasure Space in Aesthetically Important Regions: Differences between Central and Lateral Papillae. Open Dent. J..

[19] Jung J.S., Lim H.K., Lee Y.S., Jung S.K. (2024). The Occurrence and Risk Factors of Black Triangles Between Central Incisors After Orthodontic Treatment. Diagnostics.

[20] Rashid Z.J., Gul S.S., Shaikh M.S., Abdulkareem A.A., Zafar M.S. (2022). Incidence of Gingival Black Triangles following Treatment with Fixed Orthodontic Appliance: A Systematic Review. Healthcare.

[21] Rasperini G., Tavelli L., Barootchi S., McGuire M.K., Zucchelli G., Pagni G., Stefanini M., Wang H.L., Giannobile W.V. (2021). Interproximal attachment gain: The challenge of periodontal regeneration. J. Periodontol..

[22] Saleh M.H.A., Mallala D., Alrmali A., Shah B., Kumar P., Wang H.L. (2024). Residual vertical defects: Risk of disease progression, retreatment rates, and cost: A retrospective analysis. Clin. Oral Investig..

[23] Najim U., Norderyd O. (2017). Prevalence of intrabony defects in a Swedish adult population. A radiographic epidemiological study. Acta Odontol. Scand..

[24] Chow Y.C., Eber R.M., Tsao Y., Shotwell J.L., Wang H. (2010). Factors associated with the appearance of gingival papillae. J. Clin. Periodontol..

[25] Caton J.G., Armitage G., Berglundh T., Chapple I.L., Jepsen S., Kornman K.S., Mealey B.L., Papapanou P.N., Sanz M., Tonetti M.S. (2018). A new classification scheme for periodontal and peri-implant diseases and conditions—Introduction and key changes from the 1999 classification. J. Periodontol..

[26] O’Leary T.J., Drake R.B., Naylor J.E. (1972). The Plaque Control Record. J. Periodontol..

[27] Cortellini P., Prato G.P., Tonetti M.S. (1993). Periodontal Regeneration of Human Infrabony Defects. I. Clinical Measures. J. Periodontol..

[28] Farina R., Simonelli A., Minenna L., Rasperini G., Trombelli L. (2014). Single-Flap Approach in Combination with Enamel Matrix Derivative in the Treatment of Periodontal Intraosseous Defects. Int. J. Periodontics Restor. Dent..

[29] Ghezzi C., Ferrantino L., Bernardini L., Lencioni M., Masiero S. (2016). Minimally Invasive Surgical Technique in Periodontal Regeneration: A Randomized Controlled Clinical Trial Pilot Study. Int. J. Periodontics Restor. Dent..

[30] Sanz M., Herrera D., Kebschull M., Chapple I., Jepsen S., Berglundh T., Sculean A., Tonetti M.S., Merete Aass A., EFP Workshop Participants and Methodological Consultants (2020). Treatment of stage I-III periodontitis-The EFP S3 level clinical practice guideline. J. Clin. Periodontol..

[31] Levine R.A., Saleh M.H., Dias D.R., Ganeles J., Araújo M.G., Renouard F., Pinsky H.M., Miller P.D., Wang H.L. (2024). Periodontal regeneration risk assessment in the treatment of intrabony defects. Clin. Adv. Periodontics.

[32] Jeong J.S., Lee S.Y., Chang M. (2016). Alterations of papilla dimensions after orthodontic closure of the maxillary midline diastema: A retrospective longitudinal study. J. Periodontal Implant Sci..

[33] Trombelli L., Simonelli A., Minenna L., Vecchiatini R., Farina R. (2018). Simplified procedures to treat periodontal intraosseous defects in esthetic areas. Periodontology 2000.

[34] Eickholz P., Hausmann E. (2000). Accuracy of radiographic assessment of interproximal bone loss in intrabony defects using linear measurements. Eur. J. Oral Sci..

[35] Farina R., Simonelli A., Minenna L., Rasperini G., Schincaglia G.P., Tomasi C., Trombelli L. (2015). Change in the Gingival Margin Profile After the Single Flap Approach in Periodontal Intraosseous Defects. J. Periodontol..

[36] Ahmed A.J., Nichani A.S., Venugopal R. (2018). An Evaluation of the Effect of Periodontal Biotype on Inter-Dental Papilla Proportions, Distances Between Facial and Palatal Papillae in the Maxillary Anterior Dentition. J. Prosthodont..

[37] de Lemos A.B., Kahn S., Rodrigues WJde P.R., Barceleiro M.O. (2013). Influence of periodontal biotype on the presence of interdental papillae. Gen. Dent..

